# Late-onset, acquired and progressive hearing loss: surveillance and the role of the pediatrician

**DOI:** 10.1186/1824-7288-40-S1-A56

**Published:** 2014-08-11

**Authors:** Giovanni Lenzi, Stefano Berrettini, Jodi M Cutler

**Affiliations:** 1 FIMP (Italian Pediatric Federation), Grosseto, Italy; 2 University of Pisa, U.O. Otorinolaringoiatria Audiologia e Foniatria Universitaria, Pisa, Italy; 3 Affrontiamo la Sordita’ Insieme, Grosseto, Italy

## 

A survey related to universal newborn hearing screening was created with permission granted, based on the following study and adapted to the Italian situation: Primary Care Physicians’ Knowledge, Attitudes, and Practices Related to Newborn Hearing Screening (Mary Pat Moeller, PhD, Karl R. White, PhD and Lenore Shisler, MS).

First distributed in October, 2008, the survey was given to 300 pediatricians of Tuscany on a regional level, and was then adapted as an online questionnaire made available to the members of the Italian Pediatric Federation (FIMP) in April, 2009. The purpose of the survey was to verify the attitudes, experience and knowledge of family pediatricians, in regard to newborn hearing screening, hearing loss and other related arguments.

The principle objectives of the study were (1) to provide an overview of the current situation of pediatricians in regard to newborn hearing screening and follow-up; (2) to evaluate the knowledge and experience of pediatricians regarding newborn hearing screening programs and (3) to identify the information gaps in order to evaluate the most effective methods of providing the resources and information necessary to assist the families whose children are diagnosed with hearing loss.

The results indicated that there are knowledge gaps as to how to properly manage cases of children with hearing loss (for example, when and where to send newborns to specialists for necessary follow-up care, genetics and deafness, the appropriate surveillance for late-onset congenital hearing loss, as well as information on the cochlear implant and the criteria for candidacy).

In the past five years, the FIMP Audiology Network has performed Refresher courses on Newborn Hearing Screening for over 5000 pediatricians. [1]

A principle concentration on Late Onset, Acquired and Progressive Hearing Loss is now a fundamental objective.

If Pediatricians do not intervene on a national level to identify late onset hearing loss (congenital) and do not perform that which is defined as “audiological surveillance”, the screening thereby fails in its essence of being “universal”. [2] In fact, the Newborn Hearing Screening programs are not able to identify acquired or progressive hearing loss that may occur after the first months or years of life. The cases that represent an important percentage (20-30% of all cases of infant hearing loss) may only be identified through effective monitoring programs. [3]

Italian Pediatricians are in the position to guarantee this level of surveillance.

## References

[B1] White KR Status of universal newborn screening programs Paper presented at the NHS Conference 2008 Lake Como, Italy

[B2] WHO Report. Newborn and infant hearing screening Current issues and guiding principles for action: outcome of a WHO informal consultation held at WHO 2009 Geneva, Switzerland: WHO

[B3] Martini A Ipoacusie progressive di tipo genetico Acta Otorhinolaryngol ital 1998 59 Suppl 21 27 10205929

